# Socioeconomic disparities in timeliness of care and outcomes for anal cancer patients

**DOI:** 10.1002/cam4.2595

**Published:** 2019-10-09

**Authors:** Tessnim R. Ahmad, Matthew Susko, Karla Lindquist, Mekhail Anwar

**Affiliations:** ^1^ School of Medicine University of California, San Francisco San Francisco CA USA; ^2^ Department of Radiation Oncology University of California, San Francisco San Francisco CA USA; ^3^ Department of Epidemiology and Biostatistics University of California, San Francisco San Francisco CA USA

**Keywords:** anal cancer, recurrence, socioeconomic status, survival, treatment delay

## Abstract

**Background:**

Socioeconomic status (SES) is associated with diagnostic and treatment delays and survival in multiple cancers, but less data exist for anal squamous cell carcinoma (ASCC). This study investigated the association between SES and outcomes for patients undergoing definitive chemoradiation therapy for ASCC.

**Methods:**

One hundred and eleven patients diagnosed with nonmetastatic ASCC between 2005 and 2018 were retrospectively reviewed. Socioeconomic predictor variables included primary payer, race, income, employment, and partnership status. Outcomes included the tumor‐node (TN) stage at diagnosis, the duration from diagnosis to treatment initiation, relapse‐free survival (RFS), and overall survival (OS). Age, gender, TN stage, and HIV status were analyzed as covariates in survival analysis.

**Results:**

SES was not associated with the TN stage at diagnosis. SES factors associated with treatment initiation delays were Medicaid payer (*P* = .016) and single partnership status (*P* = .016). Compared to privately insured patients, Medicaid patients had lower 2‐year RFS (64.4% vs 93.8%, *P* = .021) and OS (82.9% vs 93.5%, *P* = .038). Similarly, relative to patients in the racial majority, racial minority patients had lower 2‐year RFS (53.3% vs 93.5%, *P* = .001) and OS (73.7% vs 92.6%, *P* = .008). Race was an independent predictor for both RFS (*P* = .027) and OS (*P* = .047).

**Conclusions:**

These results highlight the impact of social contextual factors on health. Interventions targeted at socioeconomically vulnerable populations are needed to reduce disparities in ASCC outcomes.

## Graphical abstract

Race and payer status were significantly associated with recurrence‐free and overall survival in patients with ASCC. Social disparities in cancer outcomes underscore the need for greater attention to the nonbiologic influencers of health in clinical care and research.

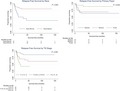

## 1 BACKGROUND

Anal cancer is a rare malignancy, with 8300 patients diagnosed each year in the United States.^1^ However, the incidence is rising due to increases in anal squamous cell carcinoma (ASCC).^1^, ^2^ ASCC is associated with human papillomavirus (HPV) infection, making it more common in men who have sex with men and patients with immunodeficiency and tobacco exposure.^3^ Patients infected with human immunodeficiency virus (HIV) are 40‐80 times more likely to develop ASCC relative to the general population.^4^

Like other HPV‐related cancers, ASCC is a preventable disease; safe sexual practices, regular screening, and, more recently, vaccination all decrease the likelihood of the infection.^5^ Low health literacy and inadequate access to these preventive measures are hypothesized to mediate higher rates of HPV infection and HPV‐associated cancers in patients of lower socioeconomic status (SES) and racial/ethnic minorities.^6^, ^7^ Socioeconomic disparities in ASCC outcomes have also been demonstrated, with low income and Black patients having a higher risk of death.^8^, ^9^

Diagnostic and treatment initiation delays have been studied as potential mediators of socioeconomic disparities in cancer outcomes. Patients who are Black, publicly insured, or living in areas with lower education have been shown to suffer longer treatment delays in ASCC.^10^ Treatment delays have been associated with decreased survival in multiple cancers.^11^, ^12^, ^13^ In addition, low SES patients have been found to present to care at more advanced stages of ASCC and other cancers, often with adverse effects on survival.^14^, ^15^, ^16^

The purpose of this study was to measure the association between SES (as measured by primary payer, race, income, employment, and partnership status) and baseline disease characteristics and outcomes. Outcomes included the tumor‐node (TN) stage at diagnosis, the duration from diagnosis to treatment initiation, relapse‐free survival (RFS), and overall survival (OS). Given the strong association between HIV infection and ASCC, this study also compared the socioeconomic characteristics of ASCC patients by HIV status.

## 2 METHODS

### 2.1 Participants

In this institutional review board‐approved study, 111 patients with biopsy‐proven, nonmetastatic ASCC treated at a large academic institution in a densely populated urban setting between 1 January 2005 and 1 May 2018 were retrospectively reviewed. All patients underwent staging with either positron emission tomography‐computed tomography (PET‐CT) or CT to rule out metastatic disease. Only patients eligible for definitive chemoradiation therapy were included. Data were obtained by reviewing patient charts in the electronic medical record and Census data using patient ZIP codes.

### 2.2 Predictor and outcome variables

Given a relatively small sample size, predictor variables were categorized into the minimum number of groups possible as follows: primary payer (private, Medicare, and Medicaid), race/ethnicity (racial majority/White, racial minority/non‐White), income (low, middle, upper), employment (employed or retired, unemployed or disabled), and partnership status (partnered, unpartnered). Incomes were estimated using median household incomes by census tract for the year during which the patient received treatment. Incomes were then categorized into three tiers by comparing the tract median household income to the surrounding metropolitan area.^17^ Retirement, as a voluntary withdrawal from prior employment accompanied by regular income such as Social Security payments and retirement account withdrawals, was combined with employed work status. Additional potentially confounding covariates between SES and baseline disease characteristics and outcomes were selected a priori and included age, gender, HIV status, and TN stage.

The primary outcomes were the TN stage at diagnosis, the duration from diagnosis to treatment initiation, RFS, and OS. Date of diagnosis was defined as the date of biopsy or, if unavailable on chart review, the date of diagnostic imaging. Date of treatment initiation was defined as the first radiation fraction date. Diagnosis to treatment initiation durations were inclusive of weekends and holidays. RFS was defined as any disease recurrence (local, regional, or distant) and death was not included. OS was defined as death due to any cause.

### 2.3 Statistical analysis

Chi‐square was used to compare the TN stage at diagnosis by SES. The duration from diagnosis to treatment initiation for each patient was log‐transformed, then means by SES were compared using the *t* test and one‐way analysis of variance (ANOVA), with pairwise comparisons for means by primary payer and income tier. For RFS and OS, survival characteristics were calculated from the date of the final radiation treatment until censoring for freedom from disease recurrence and overall survival at patients' last clinical or imaging follow‐up. RFS and OS curves were created via the Kaplan‐Meier method using the log‐rank test for significance. Unadjusted and adjusted hazard ratios for socioeconomic factors were calculated using Cox proportional hazards regression. Variables significant at *P* < .05 in univariate analysis were included in the multivariate model. All data were analyzed using STATA software version 15 (StataCorp).

## 3 RESULTS

### 3.1 Patient characteristics

Table 1 shows SES and baseline disease characteristics by payer. Compared to patients with private insurance, Medicaid patients were more likely to be unemployed or disabled (78.9% vs 7.9%, *P* < .001) and in the lowest income tier (89.5% vs 0.0%, *P* = .001). They were also more likely to be racial minorities (78.1% vs 9.4%, *P* < .001), single (57.4% vs 21.3%, *P* = .001), and HIV‐positive (66.7% vs 26.7%, *P* = .001). The TN stage at diagnosis was not associated with SES, as shown in Table 2.

**Table 1:** SES and baseline disease characteristics of ASCC patients by primary payer

SES factor or covariate	Medicaid Total (%) n = 49	Medicare Total (%) n = 21	Private Total (%) n = 34	*P*‐value
Race				
Majority	24 (33.3)	17 (23.6)	31 (43.1)	<.001
Minority	25 (78.1)	4 (12.5)	3 (9.4)	
Income level				
Low	17 (89.5)	2 (10.5)	0 (0.0)	.001
Middle	18 (43.9)	8 (19.5)	15 (36.6)	
Upper	6 (24.0)	7 (28.0)	12 (48.0)	
Employment status				
Employed or retired	14 (23.3)	16 (26.7)	30 (50.0)	<.001
Unemployed or disabled	30 (78.9)	5 (13.2)	3 (7.9)	
Partnership status				
Partnered	8 (22.9)	8 (22.9)	19 (54.2)	.001
Unpartnered	35 (57.4)	13 (21.3)	13 (21.3)	
Age, median (IQR)	56.4 (50.7‐61.1)	68.5 (64.2‐70.5)	54.0 (49.6‐58.7)	<.001
Gender				
Male	41 (59.4)	12 (17.4)	16 (23.2)	.001
Female	8 (22.9)	9 (25.7)	18 (51.4)	
TN stage				
T1/T2, N‐negative	24 (48.0)	10 (20.0)	16 (32.0)	.970
T1/T2, N‐positive and T3/T4, N‐negative	17 (43.6)	8 (20.5)	14 (35.9)	
T3/T4, N‐positive	8 (53.3)	3 (20.0)	4 (26.7)	
HIV status				
HIV‐negative	19 (32.2)	18 (30.5)	22 (37.3)	.001
HIV‐positive	30 (66.7)	3 (6.7)	12 (26.7)	

Note

Frequencies were compared between groups using Pearson's Chi‐square for categorical predictor variables and the *t* test for age.

Abbreviations: ASCC, anal squamous cell carcinoma; IQR, interquartile range; SES, socioeconomic status; TN, tumor‐node.

**Table 2:** TN stage at diagnosis for ASCC patients by SES

SES factor or covariate	T1/T2, N‐negative Total (%) n = 52	T1/T2, N‐positive and T3/T4, N‐negative Total (%) n = 42	T3/T4, N‐positive Total (%) n = 17	*P*‐value
Race				
Majority	34 (46.0)	30 (40.5)	10 (13.5)	.626
Minority	18 (48.7)	12 (32.4)	7 (18.9)	
Income level				
Low	11 (52.4)	9 (42.9)	1 (4.8)	.614
Middle	18 (40.9)	19 (43.2)	7 (15.9)	
Upper	13 (52.0)	8 (32.0)	4 (16.0)	
Employment status				
Employed or retired	28 (45.9)	24 (39.3)	9 (14.7)	.808
Unemployed or disabled	23 (52.3)	15 (34.1)	6 (13.6)	
Partnership status				
Partnered	17 (48.6)	10 (28.6)	8 (22.9)	.091
Unpartnered	32 (47.1)	30 (44.1)	6 (8.8)	
Age, median (IQR)	56.3 (50.8‐62.2)	57.5 (53.3‐63.4)	58.0 (51.1‐65.5)	.3521
Gender				
Male	34 (44.7)	31 (40.8)	11 (14.5)	.639
Female	18 (51.4)	11 (31.4)	6 (17.1)	
HIV status				
HIV‐negative	24 (40.7)	25 (42.4)	10 (16.9)	.382
HIV‐positive	28 (53.8)	17 (32.7)	7 (13.5)	

Note

Frequencies were compared between groups using Pearson's Chi‐square for categorical predictor variables and the *t *test for age.

Abbreviations: ASCC, anal squamous cell carcinoma; IQR, interquartile range; SES, socioeconomic status; TN, tumor‐node.

### 3.2 Duration from diagnosis to treatment initiation

The median duration from diagnosis to treatment initiation for the entire cohort was 7.9 weeks (IQR 5.9‐10.0). Table 3 shows median durations by SES. The median duration from diagnosis to treatment initiation was significantly longer for Medicaid patients compared to those with private insurance (8.9 weeks vs 7.4 weeks, *P* = .016). Unpartnered patients also experienced a longer delay compared to partnered patients (8.0 weeks vs 6.7 weeks, *P* = .016). No statistically significant differences were observed for unemployed compared to employed patients (8.4 vs 7.6), patients in the racial minority compared to those in the racial majority (8.1 vs 7.6), and HIV‐positive compared to HIV‐negative patients (8.7 vs 7.6).

**Table 3:** Duration (in weeks) from diagnosis to treatment initiation for ASCC patients by SES

SES factor or covariate	Median (IQR)	*P*‐value
Overall	7.9 (5.9‐10.0)	
Primary payer		
Private	7.4 (5.0‐9.9)	.029^a^
Medicaid	8.9 (6.9‐14.6)	
Medicare	7.3 (4.9‐9.4)	
Race		
Majority	7.6 (5.6‐10.1)	.254
Minority	8.1 (6.9‐10.0)	
Income level		
Low	7.6 (6.9‐8.9)	.677
Middle	7.9 (5.8‐10.0)	
Upper	7.0 (5.4‐10.0)	
Employment status		
Employed or retired	7.6 (6.0‐10.0)	.465
Unemployed or disabled	8.4 (5.6‐10.7)	
Partnership status		
Partnered	6.7 (4.6‐9.6)	.016
Unpartnered	8.0 (6.7‐11.8)	
Gender		
Male	8.0 (6.6‐10.8)	.289
Female	7.6 (5.0‐9.6)	
TN stage		
T1/T2, N‐negative	7.7 (6.7‐9.9)	.841
T1/T2, N‐positive and T3/T4, N‐negative	7.9 (5.4‐11.7)	
T3/T4, N‐positive	8.9 (5.6‐10.0)	
HIV status		
HIV‐negative	7.6 (5.6‐9.9)	.251
HIV‐positive	8.7 (6.7‐12.5)	

Note

Weeks from diagnosis to treatment initiation were log‐transformed then compared using the *t* test and one‐way analysis of variance, with pairwise comparisons for means by payer and income.

Abbreviations: ASCC, anal squamous cell carcinoma; IQR, interquartile range; SES, socioeconomic status; TN, tumor‐node.

a On pairwise comparison, *P* = .871 for private vs medicare and *P* = .016 for private medicaid.

### 3.3 Relapse‐free survival

**Figure 1:**
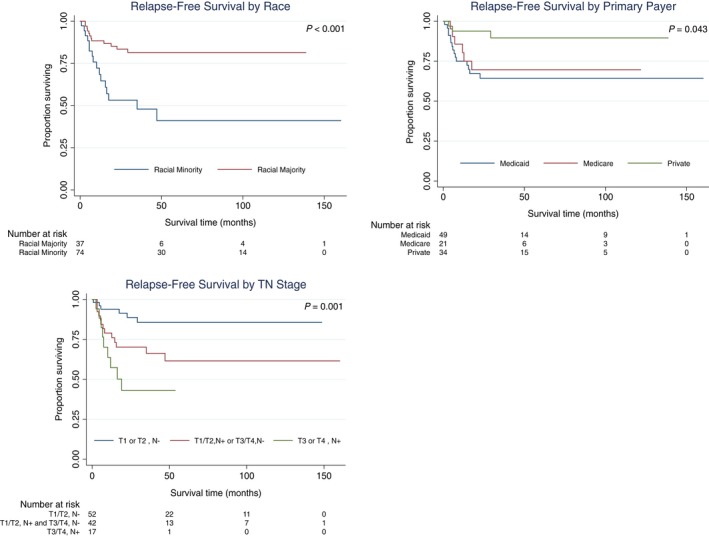
RFS in ASCC by SES. Survival curves were created in STATA software version 15 using the Kaplan‐Meier method with the log‐rank test for significance. Only variables significant at *P* < .05 in logrank test or Cox proportional hazards regression are shown. ASCC, anal squamous cell carcinoma; RFS, relapse‐free survival; SES, socioeconomic status; TN, tumor‐node

Due to overlap between the Kaplan‐Meier curves for the T1/T2, N‐positive and T3/T4, N‐negative groups, these categories were combined in order to maintain the proportional hazards assumption in the Cox models. In univariate Cox regression, race, payer, and TN stage were significantly associated with RFS. The 2‐year RFS was 64.4% for Medicaid patients compared to 93.8% for privately insured patients (HR 4.3, *P* = .021), and 53.3% for racial minorities compared to 93.5% for racial majority patients (HR 3.6, *P* = .001). Compared to T1/T2, N‐negative disease (88.7%), the 2‐year RFS was 70.2% for T1/T2, N‐positive and T3/T4, N‐negative disease (HR 4.2, *P* = .017) and 43.0% for T3/T4, N‐positive disease (HR 6.1, *P* = .001). In multivariate analysis, racial minority status (HR 2.7, *P* = .030) remained significantly associated with lower RFS, as did higher stage disease (HR 3.2, *P* = .022 for T1/T2, N‐positive and T3/T4, N‐negative; HR 4.4, *P* = .009 for T3/T4, N‐positive). Hazard ratios for relapse in univariate and multivariate Cox regression are in Table 4. Kaplan‐Meier RFS curves for significant factors are in Figure 1.

**Table 4:** SES factors associated with RFS in ASCC

SES factor or covariate	Univariate analysis			Multivariate analysis		
	HR	95% CI	*P*‐value	HR	95% CI	*P*‐value
Primary payer						
Private	Ref.			Ref.		
Medicaid	4.3	1.2‐14.9	0.021	2.9	0.8‐10.5	.110
Medicare	3.4	0.8‐13.6	0.084	3.1	0.8‐12.4	.112
Race						
Majority	Ref.			Ref.		
Minority	3.6	1.7‐7.7	0.001	2.7	1.1‐6.5	.030
Income level						
Upper	Ref.					
Low	2.5	0.7‐8.5	0.147			
Middle	1.4	0.4‐4.5	0.590			
Employment status						
Employed or retired	Ref.					
Unemployed or disabled	1.8	0.8‐3.9	0.150			
Partnership status						
Partnered	Ref.					
Unpartnered	1.7	0.7‐4.3	0.244			
Age	1.0	1.0‐1.0	0.980			
Gender						
Male	Ref.					
Female	0.6	0.2‐1.4	0.239			
TN Stage						
T1/T2, N‐negative	Ref.			Ref.		
T1/T2, N‐positive and T3/T4, N‐negative	3.1	1.2‐8.0	0.024	3.2	1.2‐8.8	.022
T3/T4, N‐positive	6.1	2.1‐17.3	0.001	4.4	1.4‐13.0	.009
HIV Status						
HIV‐negative	Ref.					
HIV‐positive	1.9	0.9‐3.9	0.099			

Note

Unadjusted and adjusted hazard ratios were calculated using Cox regression. Variables significant at *P* < .05 in univariate analysis were included in the multivariate model.

Abbreviations: ASCC, anal squamous cell carcinoma; CI, confidence interval; HR, hazard ratio; RFS, relapse‐free survival; SES, socioeconomic status; TN, tumor‐node.

### 3.4 Overall survival

**Figure 2:**
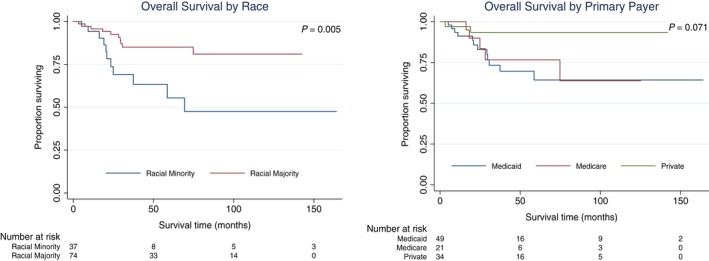
OS in ASCC by SES. Survival curves were created in STATA software version 15 using the Kaplan‐Meier method with the log‐rank test for significance. Only variables significant at *P* < .05 in log‐rank test or Cox proportional hazards regression are shown. ASCC, anal squamous cell carcinoma; OS, overall survival; SES, socioeconomic status

In univariate Cox regression, OS was significantly associated with race and payer. The 2‐year OS was 82.9% for Medicaid patients and 93.5% for privately insured patients (HR 4.9, *P* = .038). By race, the 2‐year OS was 73.7% for racial minority patients and 92.6% for racial majority patients (HR 3.2, *P* = .008). In multivariate analysis, racial minority status (HR 2.8, *P* = .047) remained significantly associated with lower OS. Hazard ratios for survival in univariate and multivariate Cox regression are in Table 5. Kaplan‐Meier curves are in Figure 2.

**Table 5:** SES factors associated with OS in ASCC

SES factor or covariate	Univariate analysis			Multivariate analysis		
	HR	95% CI	*P*‐value	HR	95% CI	*P*‐value
Primary payer						
Private	Ref.			Ref.		
Medicaid	4.9	1.1‐21.9	.038	2.9	0.6‐14.7	.189
Medicare	4.3	0.8‐22.0	.083	3.7	0.7‐19.3	.121
Race						
Majority	Ref.			Ref.		
Minority	3.2	1.4‐7.6	.008	2.8	1.0‐7.7	.047
Income level						
Upper	Ref.					
Low	2.7	0.5‐14.6	.257			
Middle	2.7	0.6‐12.6	.199			
Employment status						
Employed or retired	Ref.					
Unemployed or disabled	2.2	0.9‐5.5	.076			
Partnership status						
Partnered	Ref.					
Unpartnered	1.8	0.6‐5.5	.276			
Age	1.0	1.0‐1.0	.391			
Gender						
Male	Ref.					
Female	0.6	0.2‐1.6	.306			
TN stage						
T1/T2, N‐negative	Ref.					
T1/T2, N‐positive and T3/T4, N‐negative	2.6	1.0‐7.1	.059			
T3/T4, N‐positive	3.5	1.0‐12.8	.060			
HIV status						
HIV‐negative	Ref.					
HIV‐positive	1.5	0.6‐3.5	.353			

Note

Unadjusted and adjusted hazard ratios were calculated using Cox regression. Variables significant at *P* < .05 in univariate analysis were included in the multivariate model.

Abbreviations: ASCC, anal squamous cell carcinoma; CI, confidence interval; HR, hazard ratio; OS, overall survival; SES, socioeconomic status; TN, tumor‐node.

### 3.5 Patient characteristics by HIV status

Table 6 shows SES and baseline disease characteristics by HIV status. Compared to patients without HIV, HIV‐positive patients were significantly more likely to be male (65.8% vs 34.2%, *P* < .001) and younger (median age 54 vs 63, *P* < .001). Additionally, HIV‐positive patients were more likely to be insured by Medicaid (61.2% vs 38.8%, *P* = .001), in the racial minority (62.2% vs 37.8%, *P* = .022), in the lowest income tier (71.4% vs 28.6%, *P* = .018), and unemployed (72.7% vs 27.3%, *P* < .001).

**Table 6:** SES and baseline disease characteristics of ASCC patients by HIV Status

SES factor or covariate	HIV‐positive total (%) n = 52	HIV‐negative total (%) n = 59	*P*‐value
Race			
Majority	29 (39.2)	45 (60.8)	.022
Minority	23 (62.2)	14 (37.8)	
Income level			
Low	15 (71.4)	6 (28.6)	.018
Middle	16 (36.4)	28 (63.6)	
Upper	9 (36.0)	16 (64.0)	
Employment status			
Employed or retired	17 (27.9)	44 (72.1)	<.001
Unemployed or disabled	32 (72.7)	12 (27.3)	
Partnership status			
Partnered	14 (40.0)	21 (60.0)	.213
Unpartnered	36 (52.9)	32 (47.1)	
Primary payer			
Private	12 (35.3)	22 (64.7)	.001
Medicaid	30 (61.2)	19 (38.8)	
Medicare	3 (14.3)	18 (85.7)	
Age, median (IQR)	54.0 (49.2‐57.0)	62.7 (55.5‐68.5)	<.001
Gender			
Male	50 (65.8)	26 (34.2)	<.001
Female	2 (5.7)	33 (94.3)	
TN stage			
T1/T2, N‐negative	28 (53.8)	24 (46.2)	.382
T1/T2, N‐positive and T3/T4, N‐negative	17 (40.5)	25 (59.5)	
T3/T4, N‐positive	7 (41.2)	10 (58.8)	

Note

Frequencies were compared between groups using Pearson's Chi‐square for categorical predictor variables and the *t* test for age.

Abbreviations: ASCC, anal squamous cell carcinoma; IQR, interquartile range; SES, socioeconomic status; TN, tumor‐node.

## 4 DISCUSSION

This study investigated the impact of socioeconomic factors on the TN stage at diagnosis, treatment initiation delays, and survival in patients undergoing definitive chemoradiation therapy for ASCC. SES was not associated with the TN stage at diagnosis, but patients who were single or insured by Medicaid experienced longer delays from diagnosis to treatment initiation. Medicaid payer and racial minority status were associated with lower RFS and OS, and race was an independent predictor for both survival outcomes.

In previous studies of patients with ASCC, Black patients and patients referred from a public (as compared to a private) hospital to a radiation oncology center were found to present with more advanced disease.^14^, ^15^ Larger‐scale studies in other cancers have similarly demonstrated a correlation between SES and disease stage at presentation, with more advanced disease in non‐White, lower income, and Medicaid patients.^18^, ^19^, ^20^, ^21^ Such delays in diagnosis may primarily reflect impaired access to care, although additional socioeconomic barriers such as low health literacy regarding cancer symptomatology may also contribute.^22^, ^23^ In this study, no demographic or socioeconomic indicator included in the analysis was associated with a higher TN stage at diagnosis.

This inconsistent finding may reflect the fact that no patient lacked insurance in this study. Many treated prior to federal Medicaid expansion in 2013 were covered by Healthy San Francisco (SF), a program that subsidizes medical services for uninsured residents of the city and county of San Francisco, California. Indeed, prior studies that failed to identify an association between SES and disease stage at presentation (despite finding differences in survival by SES) were conducted in Canada and the United Kingdom, which offer universal health coverage.^24^, ^25^ Similarly, SES disparities in cancer outcomes are weak among patients in Medicare and Veterans Administration healthcare systems (large single‐payer programs).^20^, ^26^ Thus, in this study of patients residing almost exclusively in the city and county of San Francisco, SES may not serve as a proxy for access to care.^21^ It is also possible that there are patients of even lower SES who failed to present to care at all, therefore biasing this study toward patients with a threshold level of SES.

Timeliness of care is widely recognized as an important health care quality metric.^27^ ASCC patients who are Black, publicly insured, or living in areas with lower education have been shown to experience greater treatment initiation delays.^10^ In this study, median time from diagnosis to treatment initiation for the entire cohort was 7.9 weeks, with unpartnered and Medicaid patients suffering longer delays. This is consistent with research in other cancers, in which married patients have been found to present to care at earlier stages and to have improved survival.^28^ The effect of marital status may reflect increased social support and encouragement to seek and adhere to treatment.^28^ Additionally, marital status correlates with higher education and income potential and may itself be considered a component of SES.^29^ Medicaid payer status has been correlated with treatment initiation delays in other cancer sites, which may reflect socioeconomic barriers as well administrative hurdles such as longer reimbursement times and higher rates of denied claims.^22^

Socioeconomic disparities in cancer survival are well established in the research literature, persisting and even widening despite the improvements in diagnosis and treatment for many cancers.^30^ In ASCC, Black patients and patients residing in lower income areas have been shown to experience worse survival.^8^, ^9^ In this study, racial minority and Medicaid patients had significantly lower RFS and OS. The causes of socioeconomic health disparities are complex and multifactorial, which the authors conceptualize within two major categories: (a) health care system factors such as access to and quality of care, and (b) patient‐level differences in health status and cancer‐related risk factors and behaviors.

This study minimizes the first set of factors, as all patients were insured and received chemoradiation therapy at a single academic cancer center. Furthermore, the TN stage at presentation did not vary significantly by social group. The second set of factors is highly relevant, as the impact of SES on health status has been shown to be comparable in magnitude to that of well‐recognized risk factors such as diabetes and obesity.^31^ For example, in head and neck cancer, Medicaid enrollees' higher rates of alcohol and tobacco use have been shown to mediate poorer local control and overall survival.^22^ Medicaid patients also have higher rates of chronic health conditions and are more likely to rate their health negatively.^32^, ^33^ Importantly, the poor health status and outcomes of Medicaid patients may reflect the consequences of prior uninsured status, as patients may enroll in Medicaid retroactively, following a new diagnosis or catastrophic health event.^34^

Race is a robust determinant of health, with racial minorities having higher rates of illness and death.^35^ While economic factors such as income, education, and occupation trend closely with race, racial differences in health persist at all levels of SES, suggesting an independent effect of race.^35^ Indeed, in this study, race remained a significant predictor of lower RFS and OS in multivariate analysis. Exposure to psychosocial stressors such as prejudice and discrimination is hypothesized to mediate noneconomic effects of race on health.^35^

This cohort represented a high proportion of HIV‐positive patients, which is consistent with epidemiologic trends given the urban setting of this study.^36^ Compared to HIV‐negative patients, they were more likely to be racial minorities, unemployed, insured by Medicaid, and in the lowest income tier. Indeed, HIV is described as a “pandemic of the poor,” as it disproportionately affects African‐Americans, Latinos, and populations with high levels of poverty, unemployment, and psychiatric comorbidities.^37^ In this study and others, ASCC patients with HIV have not been shown to suffer worse long‐term outcomes compared to HIV‐negative patients with ASCC.^38^ However, given the clear socioeconomic vulnerabilities of this population, experts suggest that addressing the social and structural factors mediating HIV transmission is crucial to reduce rates of infection.^37^

While cancer research is advancing rapidly with newer targeted biological therapies, this study shows that SES influences cancer treatment and survival, whether as a direct contributor to outcomes or as an indicator of other related factors. We suggest that attention to the nonbiologic influencers of health—at clinical, research, and policy levels—provides an important avenue to reduce gaps in outcomes.

Clinicians may pursue such changes at the patient level or community‐ and system‐wide with Quality Improvement (QI) initiatives and policy advocacy. Considering patients' social contexts during diagnostic and therapeutic planning may reveal specific barriers to care, such as inadequate transportation, challenges in navigating cancer treatment, and lack of social support.^39^ Identification of barriers at the outset provides an opportunity for proactive, practical intervention, such as provision of transportation vouchers and consultation with a social worker. “Time to Treat” QI initiatives using patient navigators have been shown to reduce clinical delays.^40^ The use of patient navigators has also been specifically proposed as a strategy to ameliorate disparities among vulnerable populations.^41^, ^42^ Finally, via direct and longitudinal clinical care, physicians are uniquely able to identify social determinants of health. Given their credibility to the public, physicians are also positioned to influence public policy priorities in order to reduce health inequities.^43^

There are several study limitations that are important to review. This study analyzed 111 patients treated at a high‐volume academic center over 14 years. Over this period, policies affecting access to care, among many other sociopolitical factors, may have changed. Additionally, given the rarity of ASCC, treatment at a higher volume center such as our institution is likely to be associated with improved outcomes, limiting generalizability to smaller centers.^44^ Other than age and HIV status, this analysis did not account for medical comorbidities or tobacco use, the latter of which is a strong risk factor for HPV‐related cancers.^45^ Thus, the impact of SES independent of health status cannot be completely elucidated. Finally, patient incomes were estimated by census tract. San Francisco is a densely populated city and county; wealthy San Francisco neighborhoods border those plagued by poverty, and census tracts may not reflect these socioeconomic differences. Nevertheless, regardless of the precise etiology, these findings demonstrate strong social disparities in ASCC outcomes and support the need for targeted interventions aimed at marginalized populations.

## 5 CONCLUSIONS

This study of 111 ASCC patients receiving chemoradiation therapy at a single academic medical center demonstrates that SES is significantly associated with treatment delays, RFS, and OS. These findings underscore the importance of social contextual factors in ASCC outcomes. Greater attention in clinical practice and research to the nonbiologic influencers of health is needed to improve outcomes in socioeconomically vulnerable populations.

## CONFLICT OF INTEREST

No authors have conflict of interest to declare.

## AUTHOR CONTRIBUTIONS

All authors contributed to the study design, data analysis, and writing of the manuscript, with the first author assuming primary responsibility. The second and senior authors compiled the original clinical dataset.
